# Mapping UK mental health services for adults with attention-deficit/hyperactivity disorder: national survey with comparison of reporting between three stakeholder groups

**DOI:** 10.1192/bjo.2020.65

**Published:** 2020-07-29

**Authors:** Anna Price, Astrid Janssens, Tamsin Newlove-Delgado, Helen Eke, Moli Paul, Kapil Sayal, Chris Hollis, Cornelius Ani, Susan Young, Susan Dunn-Morua, Philip Asherson, Stuart Logan, Tamsin Ford

**Affiliations:** College of Medicine and Health, University of Exeter, UK; Department of Public Health, University of Southern Denmark, Odense, Denmark; and College of Medicine and Health, University of Exeter, UK; College of Medicine and Health, University of Exeter, UK; College of Medicine and Health, University of Exeter, UK; Division of Mental Health and Wellbeing, Warwick Medical School, University of Warwick; and Coventry & Warwickshire Partnership, UK; Faculty of Medicine and Health Sciences, University of Nottingham, UK; School of Medicine, University of Nottingham; National Institute for Health Research (NIHR) MindTech and in Vitro Diagnostics Co-operative (MIC), Faculty of Medicine & Health Sciences; and NIHR Nottingham Biomedical Research Centre, Nottingham, UK; Department of Brain Sciences, Faculty of Medicine, Imperial College London; and Surrey and Borders Partnership NHS Foundation Trust, UK; Department of Psychology, Psychology Services Ltd, London, UK; and University of Reykjavik, Iceland; AADD–UK, Bristol, UK; Department of Social Genetic & Developmental Psychiatry, Institute of Psychiatry, King's College London, UK; College of Medicine and Health, University of Exeter; NIHR Applied Research Collaboration South West Peninsula (NIHR PenARC), University of Exeter; and Royal Devon and Exeter Foundation NHS Trust, UK; Department of Psychiatry, University of Cambridge, UK

**Keywords:** ADHD, survey, health services, stakeholders, UK

## Abstract

**Background:**

UK clinical guidelines recommend treatment of attention-deficit hyperactivity disorder (ADHD) in adults by suitably qualified clinical teams. However, young people with ADHD attempting the transition from children's to adults’ services experience considerable difficulties in accessing care.

**Aims:**

To map the mental health services in the UK for adults who have ADHD and compare the reports of key stakeholders (people with ADHD and their carers, health workers, service commissioners).

**Method:**

A survey about the existence and extent of service provision for adults with ADHD was distributed online and via national organisations (e.g. Royal College of Psychiatrists, the ADHD Foundation). Freedom of information requests were sent to commissioners. Descriptive analysis was used to compare reports from the different stakeholders.

**Results:**

A total of 294 unique services were identified by 2686 respondents. Of these, 44 (15%) were dedicated adult ADHD services and 99 (34%) were generic adult mental health services. Only 12 dedicated services (27%) provided the full range of treatments recommended by the National Institute for Health and Care Excellence. Only half of the dedicated services (55%) and a minority of other services (7%) were reported by all stakeholder groups (*P* < 0.001, Fisher's exact test).

**Conclusions:**

There is geographical variation in the provision of NHS services for adults with ADHD across the UK, as well as limited availability of treatments in the available services. Differences between stakeholder reports raise questions about equitable access. With increasing numbers of young people with ADHD graduating from children's services, developing evidence-based accessible models of care for adults with ADHD remains an urgent policy and commissioning priority.

The UK National Institute for Health and Care Excellence (NICE) guidelines state that the following services should be available for adults with attention-deficit hyperactivity disorder (ADHD): transitional care, assessment and diagnostic services, medication titration, monitoring and review, and psychological treatments.^1^ NICE also recommends that treatment should be holistic and provided by multidisciplinary teams or clinicians with expertise in ADHD, with shared care protocols with primary care in place after medication titration and dose stabilisation.^1^ Shared care is defined as the planned joint participation of consultants and general practitioners (GPs) in the delivery of care for patients with a chronic condition.^2^ Although there are effective, evidence-based treatments for adults who have ADHD,^3^ there is no consensus about the optimum organisation of health services to provide them.^4^ Mounting evidence suggests that, despite evidence-based treatments, guideline recommendations are frequently ignored, so that adults with ADHD struggle to access appropriate healthcare.^5^ A recent systematic review found that a lack of available information about services for adults with ADHD created difficulties for both referring clinicians and patients accessing treatment.^6^ People with ADHD are already at increased risk of poor health, social, educational and occupational outcomes, and without access to appropriate healthcare they face higher risks of negative outcomes, including substance misuse, criminality and road traffic accidents.^5,7–9^ As increasing numbers of young people with ADHD graduate from children's services, providing national information about adult services and investigating access to care are priorities.

At the time we undertook this study (January–February 2018), there was limited research and grey literature about the provision of services for adults with ADHD across the UK. Studies reported in the literature either covered a specific region or described young people's experiences of transition, rather than mapping the services available for young people with ADHD transitioning to mental health services for adults.^10–14^ In addition, studies of service availability have tended to draw on the perspectives of one type of stakeholder, such as senior healthcare professionals (not working in frontline services)^14^ or healthcare professionals working in child or adult health services,^10^ rather than including perspectives of senior healthcare staff, frontline staff, commissioners and patients. Surveying a range of key stakeholders minimises the likelihood that a service will be overlooked, while comparison of their reports provides important information about gaps in awareness among different groups.

As recommended by Hall et al in 2013, the study reported in this paper aimed to provide national-level data on UK mental health service provision for adults with ADHD.^10^ We aimed to provide: a geographical overview of services; details of treatment provided by dedicated National Health Service (NHS) adult ADHD services; and an exploration of differences in reports of services by key stakeholder group (commissioners, health workers and service users).

## Method

This work formed part of the Children and Adolescents with ADHD in Transition from Child to Adult Services (CATCh-uS) study of transition in ADHD.^15^ We assert that all procedures contributing to this work comply with the ethical standards of the relevant national and institutional committees on human experimentation and with the Helsinki Declaration of 1975, as revised in 2008. All procedures involving human participants were approved by the University of Exeter Medical School Ethics Committee (research ethics committee application number: 15/07/070). Following consultation with the research ethics committee, a statement on confidentiality and data usage was included at the beginning of the survey, with the understanding that, by continuing with the survey, participants were providing informed consent for the planned anonymous use of their data.

The novel mapping methodology was developed iteratively, with extensive patient and public involvement and is reported in full elsewhere.^16^ The definitive study is described below.

### Participants

Our sample frame was all stakeholders involved in the care process for young people needing transition from child to adult services, as well as those involved in allocating and financing local services. This included young adults with ADHD and their parents/carers, members of clinical teams (such as psychiatrists, paediatricians, psychologists, GPs, nurses, practice managers, administrators) and service commissioners (clinical commissioning groups in England, health boards in Scotland and Wales, and health and social care trusts in Northern Ireland).

### Sampling strategy

Informants were purposively sampled from three key stakeholder groups (service users, healthcare workers and commissioners) via multiple methods. Three data sources informed the service map: a national online survey, freedom of information (FOI) requests and a surveillance study.

#### Anonymous national online survey (convenience sample)

Links to an online survey were shared with stakeholders via emails from organisational mailing lists, newsletters and websites, and through social media. A snowballing technique was used to recruit additional stakeholders and their organisations. The survey was open for 5 weeks from January 2018.

#### Freedom of information requests (total population)

Organisations responsible for commissioning, or planning and funding, NHS mental health services in the UK were sent survey questions via FOI requests in January 2018. These are legal processes that support the rights of people to gain access to information that is recorded and held by public-sector organisations.^17^ A copy of the survey, examples of FOI requests made and a list of key supporting organisations are provided in the supplementary material, available at https://doi.org/10.1192/bjo.2020.65.

#### Surveillance (purposive sample)

Reports of transition in ADHD services were collated from paediatricians and psychiatrists who responded to a national surveillance study on young people in need of a transition into adult services. This was run via the British Paediatric Surveillance Unit and the Child and Adolescent Psychiatry Surveillance System from December 2016 for 12 months. Reported cases were followed up after 9 months (August 2017 to August 2018).

### Data collection

#### Survey

The brief online survey, hosted by Survey Monkey, consisted of between five and nine questions, depending on user responses. It collected basic demographic information, including respondents’ locations (postcode or region in the UK) and respondents’ links with ADHD (e.g. ‘adult with ADHD’ or ‘psychiatrist’), then asked for details of services they had knowledge of for adults with ADHD. Services were broadly defined as ‘any mental health service for people with ADHD aged 18 and above’, with notes clarifying that this could include any ‘specialist doctor or team, mental health team, clinic, charity or support group that treats or supports adults with ADHD’. Respondents identified services from a pre-populated list and could identify services that were not already listed. For every service they identified, respondents were asked to confirm whether it was somewhere that they, or someone they knew of, had ‘received treatment or support […] for their adult ADHD’.

FOI requests collected basic demographic information on the commissioning organisation and asked whether they commissioned ‘mental health services that treat/support people with ADHD aged 18 years and above’. If yes, they were asked to provide details of the services that were similar to the details requested in the survey, as well as to specify the type of service and which treatments were available.

For all NHS-provided dedicated adult ADHD services (group A; see definition below), details of provision were also checked via FOI requests to the provider (details in the supplementary material).

#### Surveillance study

The CATCh-uS national surveillance study collected data from child and adolescent psychiatrists and paediatricians on transition outcomes of young adults with ADHD.^18^ Reports of services from this study were triangulated with services already mapped, with the intention of incorporating additional services, if any were reported.

### Data analysis

#### Sample

Informants were categorised into three main stakeholder groups (service user, commissioner or health worker), depending on their strongest link with ADHD. For example, a parent/carer/partner of someone with ADHD was categorised as a service user, while psychiatrists were categorised as health workers. Descriptive statistics summarised respondents’ characteristics by data source, geographic location and stakeholder group (service user, commissioner or health worker). Given the non-probabilistic sampling frame, a pragmatic minimum target of 50 informants per UK NHS region was identified to ensure adequate coverage.

#### Data cleaning

Raw data on services were matched against existing online information by A.P. and checked at least once by other members of the research team. Where details could not be matched to an existing service, they were independently checked a minimum of three times before being categorised as unidentifiable.

#### Services identified

All of the identified services were recorded. Services for which at least one respondent had confirmed experience of treatment for their ADHD as an adult were categorised into the following three groups:

- NHS dedicated services for adults with ADHD
- NHS non-dedicated services for adults with ADHD
- other services that work with adults with ADHD (including NHS provision for children, charity/voluntary and private).

Services were defined as dedicated if they had ‘ADHD’ or ‘neurodevelopmental’ in the service name. The term ‘dedicated’ was used rather than ‘specialist’ so that generic NHS services with named clinics with dedicated time for adults with ADHD would be included. Service locations were plotted on a map of the UK, using QGIS 2.18 for Windows^19^ and uploaded onto a Google My Map to provide a visual summary of service availability and to communicate findings with stakeholders. The balance of responses by UK region and stakeholder group was similarly mapped.

#### Stakeholder perspectives

For each service, a descriptive summary was created of the stakeholder groups, and combinations of stakeholder groups, that had identified that service. The percentages of services identified by stakeholder group, and for each service type, were summarised and tabulated. The association between stakeholder type and service reporting was tested using Pearson's χ^2^. Differences between combinations of stakeholders reporting services were tested using Fisher's exact test, and overlap was displayed using Venn diagrams.

## Results

### Informants

In total, 2686 reports of services were included in the study: 73% (*n* = 1946) were from health workers, 17% (*n* = 461) from service users, 8% (*n* = 216) from commissioners and 2% (*n* = 63) from others such as educational practitioners or researchers.

**Fig. 1:**
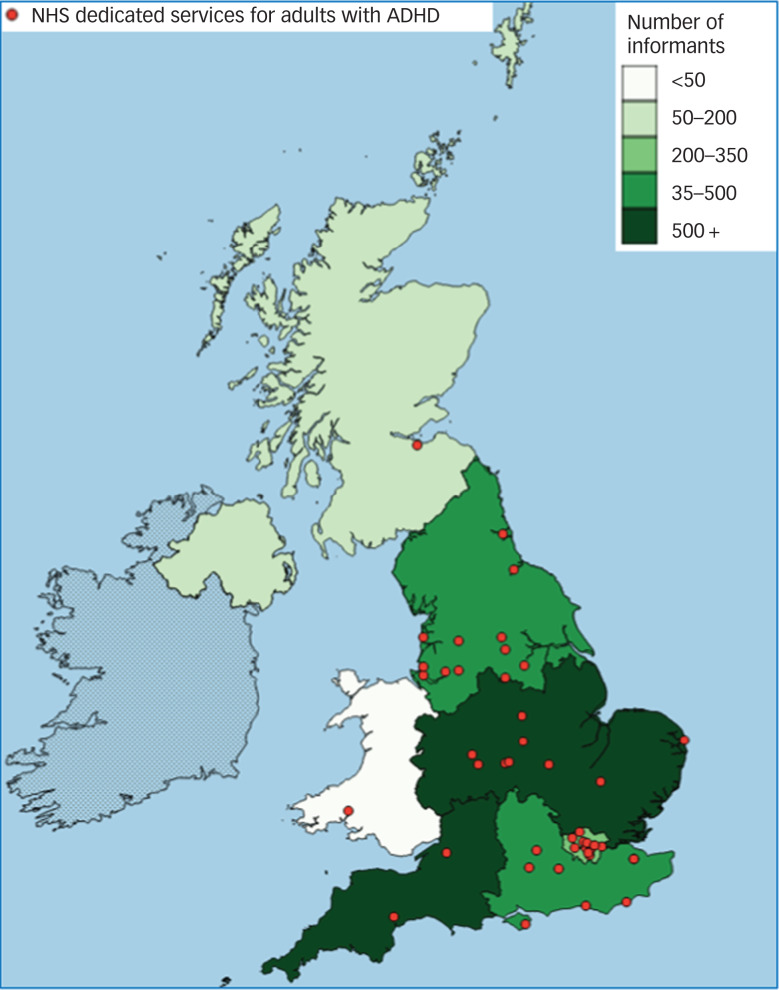
Numbers of mapping study informants per National Health Service (NHS) region, and the locations of the 44 NHS dedicated services for adults with attention-deficit hyperactivity disorder (ADHD) in the UK (group A), as identified by study informants.

Most reports of services (*n* = 2158, 80%) were obtained from the online survey, compared with commissioners responding to FOI requests (*n* = 213, 8%) and the surveillance study (*n* = 315, 12%). Of the 236 organisations sent FOI requests, 213 (90%) responded. Response rates to questionnaires for the surveillance study were also high (79% at baseline, 82% at follow-up). The minimum of 50 informants per NHS region report was reached for every region except Wales, where 40 reports were received. For a geographic overview of the locations of informants, see Fig. 1. A more detailed breakdown of the sample by data source and stakeholder identity is available in the CATCh-uS study report.^20^

### Services

In total, 294 unique services were identified, with 254 services for which informants confirmed experience of treatment or support for an adult with ADHD (Table 1.)

**Table 1:** Services for adults with attention-deficit hyperactivity disorder (ADHD) by service group and service type

Services for adults with ADHD, by service group	Service type	Service types, *n*	Total by service group, *n* (%)
Group A: NHS dedicated services for adults	NHS adult ADHD	29
	NHS adult ADHD and ASD	7
	NHS adult neurodevelopmental	8	44 (17)
Group B: NHS non-dedicated services for adults	NHS 0–25 year-olds	2
	NHS adult ASD	2
	NHS adult drug and alcohol	1
	NHS AMH CMHT	70
	NHS health and social care	1
	NHS adult learning disability	17
	NHS AMH and learning disability	2
	NHS AMH primary care	2
	NHS AMH prison and custody	2	99 (39)
Group C: Other services for adults with ADHD	Charity/Voluntary	15
	Charity/Voluntary (support group)	24
	NHS child ADHD specialist	3
	NHS child neurodevelopmental	3
	NHS generic child	26
	Private	36
	Private (social enterprise)	4	111 (44)

NHS, National Health Service; ASD, autism spectrum disorder; AMH, adult mental health; CMHT, community mental health team; learning disability, the NHS term for intellectual disability; child, child and adolescent mental health or paediatric service (for under 18-year-olds).

#### Dedicated services

Responses to FOI requests checking details of provision at the 44 NHS dedicated services for adults with ADHD (group A) were received from 89% (31/35) of providing organisations. Responses indicated that only 12 services (27%) offered the range of interventions specified by NICE.^1^ Services were most likely to offer medication management, shared care or ongoing prescribing (*n* = 39, 89%) and diagnostic assessment (*n* = 36, 82%); psychological treatment (*n* = 22, 50%) and transitional care (*n* = 26, 59%) were less frequently reported. Two services (5%) reported an upper age limit of 65 years, and almost one-third (*n* = 13, 30%) reported that patients from outside their commissioned area might be able to access treatments in that service. Figure 1 illustrates the uneven distribution of NHS dedicated services for adults with ADHD across the UK.

### Stakeholder perspectives

**Fig. 2:**
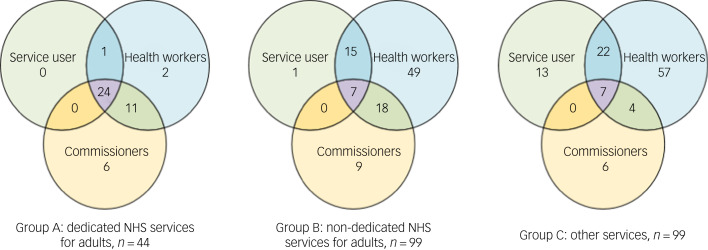
Venn diagrams illustrating the combinations of stakeholder groups identifying services for adults with attention-deficit hyperactivity disorder. The service user group includes both adults with ADHD and parents/carers/partners of someone with ADHD.

Table 2 provides a descriptive summary of service reporting by stakeholder group and combination of stakeholder groups, and Fig. 2 indicates the overlap, or lack thereof, between their reports of different levels of service provision.

**Table 2:** Numbers of services identified by stakeholder groups (service users, health workers and commissioners) and combinations of stakeholder groups identifying services, by service group

Service group	Services, *n*	Services identified by each stakeholder group, *n* (%)			Services as identified by different combinations of stakeholder groups, *n* (%)							
		Service users	Health workers	Commissioners	All three groups	Service users and health workers	Service users and commissioners	Health workers and commissioners	Service users	Health workers	Commissioners	Other
Group A	44	25 (57)	38 (86)	41 (93)	24 (55)	1 (2)	0 (0)	11 (24)	0 (0)	2 (5)	6 (14)	0 (0)
Group B	99	23 (23)	89 (90)	34 (34)	7 (7)	15 (15)	0 (0)	18 (18)	1 (1)	49 (50)	9 (9)	0 (0)
Group C	111	42 (38)	90 (81)	17 (15)	7 (6)	22 (20)	0 (0)	4 (4)	13 (12)	57 (51)	6 (5)	2 (2)

Service groups: A, NHS dedicated adult; B, NHS non-dedicated adult; C, NHS non-adult, and private and voluntary.

There were significant differences in the proportions of NHS dedicated adult (group A), NHS non-dedicated adult (group B) and other (group C) services reported by each stakeholder group (χ^2^(4,399) = 34.29, *P* < 0.001). Service users were marginally more likely to report group A or group C services, and less likely to report group B services (χ^2^(2,344) = 7.13, *P* = 0.03). Health workers reported similar proportions of group A, B and C services (χ^2^(2,471) = 0.26, *P* = 0.88). Commissioners were more likely to report group A services than group B or C (χ^2^(4,399) = 34.29, *P* < 0.001).

As Fig. 2 illustrates, a higher proportion of NHS dedicated adult (group A) services was reported by all stakeholder groups (*n* = 24, 55%), compared with NHS non-dedicated adult (group B) services (*n* = 7, 7%) and other (group C) services (*n* = 7, 6%; *P* < 0.001, Fisher's exact test). The majority of NHS non-dedicated adult (group B) and other (group C) services were reported by health workers alone.

## Discussion

The study reported in this paper makes a unique contribution to the research literature by presenting national-level data about the services for adults who have ADHD that were available in the UK in 2018. It highlights geographical gaps in NHS services in the UK for adults who have ADHD, shows that service provision is limited, and documents major differences in different groups of stakeholders’ awareness of the services that are available. In doing so, it updates, supports and extends the existing evidence of patchy provision.^10,11,13,14^ The significant differences in the types of services identified by stakeholders raise questions about equitable access to care for adults with ADHD, particularly in areas without dedicated services.

### Service types

Gaining a clear picture of provision was not straightforward because of differences in NHS service organisation by country and region of the UK. For example, many health services in England are funded via commissioning bodies, whereas in other countries, such as Wales, the commissioning process is often described instead as planning and financing, with the agencies responsible the same as those responsible for service delivery. Differences in the structures of the respective National Health Services of each country in the UK, and how they function across the four jurisdictions, may have influenced the number of responses we had, as well as the way that services were reported.

Informants reported experiences of treatment for adults with ADHD at a range of service types. However, only 44 were ‘dedicated’ NHS services for adults (those with ‘ADHD’ or ‘neurodevelopmental’ in their name) and, of these, less than one-third offered the full range of treatments recommended by the NICE guidelines.^1^ Although treatment with medication was available at more than 80% of dedicated services, psychological treatment was available at only half. It is possible that the treatments recommended by NICE that were not available at some dedicated services were provided by other local NHS services. This seems to us to be unlikely, given that previous research suggests that patients struggle to access the full range of recommended services, including support for young people who are transitioning between services, and psychological treatments.^6,18,21^ Dedicated services did not indicate to us that they sourced these treatments for patients elsewhere, but further research could explore this explicitly.

Owing to the current complexity of the organisation of services for adults with ADHD in the UK, it was difficult to assess whether a lack of a dedicated service equated to the lack of any commissioned service for adults with ADHD in that area. Existing evidence suggests that young people with ADHD may not meet referral criteria for generic adult mental health services, and there can be difficulties in accessing treatment related to a lack of training and specialist knowledge among staff.^4,6^ Some stakeholders would argue that UK regions with no ‘dedicated’ services represent a gap in provision of care for adults with ADHD. Although an additional 99 ‘non-dedicated’ UK adult NHS services were identified, their existence was most commonly reported only by health workers, rather than service users or commissioners. This suggests that these services may be less accessible, with possible implications for resourcing. Existing qualitative research suggests that service users may be more satisfied with the care for adults who have ADHD that they received at dedicated or ‘specialist’ services.^22^

A surprisingly high number of ‘other services’ were identified at which support had been experienced, including child NHS services, private and charitable services. These may represent additional choice and a richer variety of healthcare options, although this needs consideration in the context of difficulties faced by patients trying to access appropriate NHS care for adult ADHD.^23^ Previous research suggests that clinicians who work in NHS-provided services for children may continue to deliver treatment beyond the upper age specified for their service in locations where no service for adults is available. This may have an impact on the capacity to respond to younger children in need.^21^ There are also reports of adults seeking privately funded healthcare when no other route to treatment is available,^24^ and 40 such services were reported in the present study, highlighting potentially significant out-of-pocket expenses incurred by people with ADHD. This raises concerns for the well-being of the most vulnerable members of the population for whom private healthcare is not an option and who lack advocates to negotiate or navigate services on their behalf.

There is still no clear consensus on optimal models for the provision of care for adults with ADHD;^4^ the NICE guidelines state only that a service should be provided by teams of ‘healthcare professionals with training and expertise in diagnosing and managing ADHD’.^1^ Future research should explore different models of service provision within primary and secondary healthcare services, including evaluations of their effectiveness and cost-effectiveness. There is also scope for further mapping to explore the uptake and availability of shared care for ADHD, as qualitative research suggests that some young adults are treated exclusively by their GPs, while others experience difficulties finding a GP willing to prescribe medication even under shared care arrangements.^20^ This suggests that the implementation of shared care arrangements may be highly variable.

### Strengths and limitations

This research has provided the most extensive data to date about the availability of services for adults in the UK who have ADHD, and it extends existing region-specific and single-source information^10,14^ by triangulating reports from a range of stakeholders. The use of FOI requests to contact commissioners ensured that staff with time and resources responded to enquiries, and proved effective, with a 90% response rate. The novel survey methodology, including collaboration with partner organisations, was a rapid and effective way of gathering reports from a range of stakeholders across the UK.^16^ However, although a target minimum number of responses was received from all but one UK region, the use of non-probabilistic sampling methods meant that respondents were not selected randomly. Necessarily, informants would have been computer literate and interested in ADHD. It is possible that this introduced bias, with survey informants more likely to be those who had struggled to access healthcare. The use of multiple informants and methods, combined with the high number of responses, mitigated the risk of bias and made it likely that the vast majority of relevant services were identified.

A clearer definition of ‘dedicated’ services would have improved the quality of the service map. However, given the complexity of health service provision in the UK, which made it difficult to be sure that health workers, service users and commissioners were identifying the same unit of ‘service’ when responding to the survey, we chose our definition to ensure that specialist teams and those generic services with practitioners with dedicated time to focus on adults with ADHD could be included on the map. The methodological decision to label services as dedicated meant that identified services comprised a range, from highly specialist national and regional services to clinicians with only a few days a month dedicated to ADHD-related work within their generic adult mental health service. Resource limitations meant that service details were checked only with providers of dedicated services, and their capacity, in terms of staffing levels, and key indicators such as waiting-list times were not evaluated. During analysis, differences in service organisation by country and region of the UK made it difficult to ascertain whether an area without a dedicated service was also therefore an area without a commissioned service for adults with ADHD. Findings from the analysis of differences in reporting should be considered in the context of the balance of survey responses, with the majority of responses coming from health workers. As UK health services for adults with ADHD are continually evolving, this research provides only a snapshot in time. However, this baseline map of services has been hosted by the UK Adult ADHD Network (https://www.ukaan.org/adult-adhd-service-map), which will maintain and update it over time, so that it is a useful resource for all stakeholders.

### Implications

Given the importance of continuing treatment for ADHD into adulthood where needed,^5,7,8^ the increasing numbers of young people with ADHD graduating from child services and the existence of effective evidence-based treatments,^3^ these data highlight the urgent need to improve provision and access for this vulnerable population. Clearly defined, accessible and equitable services for adults with ADHD are needed, combined with better information about what is available for public and professionals. The map of services is a tangible resource to provide better quality and accessible information to all stakeholders, the lack of which has been identified as a barrier when patients need to transition into adult services.^6^

The geographic gaps in the availability of NHS dedicated services for adults with ADHD, as well as limited availability of the treatment options recommended in the NICE guidelines, suggests that where someone lives will have an impact on whether or not appropriate treatment is available to them, which is contrary to the stated aim of the NHS of equitable access to appropriate healthcare for people with long-term conditions, and should be addressed as a matter of urgency.

## Data Availability

The data are currently stored securely by the University of Exeter Medical School, under embargo until the end of the CATCh-uS project.

## Supplementary material

For supplementary material accompanying this paper visit http://dx.doi.org/10.1192/bjo.2020.65. click here to view supplementary material

## References

[1] National Institute for Health and Care Excellence. Attention Deficit Hyperactivity Disorder: Diagnosis and Management (NICE Guideline NG87). NICE, 2018. 29634174

[2] Hickman H, Drummond N, Grimshaw J. The operation of shared care for chronic disease. Health Bull-Scott Home Health Dep 1994; 52: 118–26. 10.1093/oxfordjournals.pubmed.a043026 7880576

[3] Bolea-Alamanac B, Nutt DJ, Adamou M, Asherson P, Bazire S, Coghill D, Evidence-based guidelines for the pharmacological management of attention deficit hyperactivity disorder: update on recommendations from the British Association for Psychopharmacology. J Psychopharmacol 2014; 28: 179–203. 24526134 10.1177/0269881113519509

[4] Coghill D. Organisation of services for managing ADHD. Epidemiol Psych Sci 2017; 26: 453–8. 10.1017/S2045796016000937 PMC6998894 28004618

[5] Kooij JJS, Bijlenga D, Salerno L, Jaeschke R, Bitter I, Balazs J, Updated European Consensus Statement on diagnosis and treatment of adult ADHD. Eur Psychiatry 2019; 56: 14–34. 30453134 10.1016/j.eurpsy.2018.11.001

[6] Price A, Janssens A, Woodley AL, Allwood M, Ford T. Review: experiences of healthcare transitions for young people with attention deficit hyperactivity disorder: a systematic review of qualitative research. Child Adolesc Ment Health 2019; 24: 113–22. 32677182 10.1111/camh.12297

[7] Lichtenstein P, Halldner L, Zetterqvist J, Sjolander A, Serlachius E, Fazel S, Medication for attention deficit-hyperactivity disorder and criminality. N Engl J Med 2012; 367: 2006–14. 23171097 10.1056/NEJMoa1203241 PMC3664186

[8] Chang Z, Lichtenstein P, D'Onofrio BM, Sjolander A, Larsson H. Serious transport accidents in adults with attention-deficit/hyperactivity disorder and the effect of medication: a population-based study. JAMA Psychiatry 2014; 71: 319–25. 24477798 10.1001/jamapsychiatry.2013.4174 PMC3949159

[9] Young S, Gudjonsson G, Chitsabesan P, Colley B, Farrag E, Forrester A, Identification and treatment of offenders with attention-deficit/hyperactivity disorder in the prison population: a practical approach based upon expert consensus. BMC Psychiatry 2018; 18(1): 281. 30180832 10.1186/s12888-018-1858-9 PMC6122636

[10] Hall CL, Newell K, Taylor J, Sayal K, Swift KD, Hollis C. ‘Mind the gap'–mapping services for young people with ADHD transitioning from child to adult mental health services. BMC Psychiatry 2013; 13: 186. 23842080 10.1186/1471-244X-13-186 PMC3717001

[11] Edwin F, McDonald J. Services for adults with attention-deficit hyperactivity disorder: national survey. Psychiatric Bulletin 2007; 31: 286–8.

[12] Taylor N, Fauset A, Harpin V. Young adults with ADHD: an analysis of their service needs on transfer to adult services. Arch Dis Child 2010; 95: 513–7. 20530525 10.1136/adc.2009.164384

[13] Zaman R, Arif M, Vaze A, Müller U. Setting up adult ADHD service in the United Kingdom. Cutting Edge Psychiatry Pract 2012; 1: 170–5.

[14] Hall CL, Newell K, Taylor J, Sayal K, Hollis C. Services for young people with attention deficit/hyperactivity disorder transitioning from child to adult mental health services: a national survey of mental health trusts in England. J Psychopharmacol 2015; 29: 39–42. 25237121 10.1177/0269881114550353

[15] Ford T, Janssens A, Asherson P, Beresford B, Paul M, Ani C, CATCh-uS: Children with ADHD in Transition from Children's Services to Adult Services (Protocol 23 Oct 2015). NIHR, 2015 (https://www.journalslibrary.nihr.ac.uk/programmes/hsdr/142152/#/documentation).

[16] Price A, Janssens A, Dunn-Morua S, Eke H, Asherson P, Lloyd T, Seven steps to mapping health service provision: lessons learned from mapping services for adults with Attention-Deficit/Hyperactivity Disorder (ADHD) in the UK. BMC Health Serv Res 2019; 19: 468. 10.1186/s12913-019-4287-7 PMC6617903 31288805

[17] Information Commissioners Office. The Guide to Freedom of Information 2016. ICO, 2018.

[18] Eke H, Ford T, Newlove-Delgado T, Price A, Young S, Ani C, Transition between child and adult services for young people with attention-deficit hyperactivity disorder (ADHD): findings from a British national surveillance study. Br J Psychiatry [Epub ahead of print] 4 Jun 2019 Available from: 10.1192/bjp.2019.131. PMC7589988 31159893

[19] QGIS Development Team. QGIS Geographic information system, version 2.18. Open Source Geospatial Foundation Project. QGIS, 2018.

[20] Janssens A, Eke H, Price A, Newlove-Delgado T, Blake S, Ani C, *Young people with Attention Deficit Hyperactivity Disorder (ADHD) in transition from children's services to adult services (Catch-uS): a mixed methods project using national surveillance, qualitative and mapping studies.* Health Services and Delivery Research [cited 29 Mar 2019]. Available from: https://www.journalslibrary.nihr.ac.uk/programmes/hsdr/142152/#/.

[21] Young S, Adamou M, Asherson P, Coghill D, Colley B, Gudjonsson G, Recommendations for the transition of patients with ADHD from child to adult healthcare services: a consensus statement from the UK adult ADHD network. BMC Psychiatry 2016; 16: 301. 27561259 10.1186/s12888-016-1013-4 PMC5000407

[22] Matheson L, Asherson P, Wong IC, Hodgkins P, Setyawan J, Sasane R, Adult ADHD patient experiences of impairment, service provision and clinical management in England: a qualitative study. BMC Health Serv Res 2013; 13: 184. 23692803 10.1186/1472-6963-13-184 PMC3665585

[23] Belling R, McLaren S, Paul M, Ford T, Kramer T, Weaver T, The effect of organisational resources and eligibility issues on transition from child and adolescent to adult mental health services. J Health Serv Res Policy 2014; 19: 169–76. 24700210 10.1177/1355819614527439

[24] Wong IC, Asherson P, Bilbow A, Clifford S, Coghill D, DeSoysa R, Cessation of attention deficit hyperactivity disorder drugs in the young (CADDY): a pharmacoepidemiological and qualitative study. Health Technol Assess 2009; 13(50): iii–120. 10.3310/hta13490 19883527

