# Supplementary material for: Mapping UK mental health services for adults with attention-deficit/hyperactivity disorder: national survey with comparison of reporting between three stakeholder groups
Source: BJPsych Open. 2020 Jul 29;6(4):e76. doi: 10.1192/bjo.2020.65 (PMC7443899; doi:10.1192/bjo.2020.65)
Supplement: Supplementary file 1 [file S2056472420000654sup001.zip › S2056472420000654sup001.docx]

**Subject**: Freedom of information request - CATCh-uS 2018 National survey mapping adult ADHD services

**Text:**

Dear Sir/Madam

Please provide the following information about services provided by your Trust* for adults (people aged 18 and above) with Attention Deficit Hyperactivity Disorder (ADHD).

*If you provide FOI for more than one MHT, please answer questions separately for **every** MHT you represent. This is a national survey so we want to know about all MHTs in England and we have only contacted FOI email addresses once, to avoid duplication of workload.

**2018 Survey for the ‘Children and adolescents with Attention Deficit Hyperactivity Disorder in transition between children’s services and adult services’ (CATCh-uS) study.**

The CATCh-uS study is funded by the National Institute for Health Research and has ethical approval. Details can be found on our [website](http://medicine.exeter.ac.uk/catchus/) (<http://medicine.exeter.ac.uk/catchus/>). Answers will help to update a list of existing services, available here (<http://medicine.exeter.ac.uk/catchus/mapping/adhdservices/>).

Thank you in advance for your support.

| **Part 1: Overview** |
| --- |
| Which Health Trust (MHT) do you represent?   - 1. Name:   2. Postcode: |
| Who is responsible for provision of adult ADHD mental health services in your trust? (e.g. lead for mental health services or head of department)   - 1. Name:   2. Email Address:   3. Job Role: |
| Which NHS England region is your Trust part of?   - 1. London   2. Midlands and East of England   3. North of England   4. South West England   5. South East England |
| Which region of England is your trust in?   - 1. East Midlands   2. Eastern   3. London   4. North East   5. North West   6. South East   7. South West   8. West Midlands   9. Yorkshire and the Humber |
| Does your Trust provide services for people with ADHD aged 18 years and above?   - 1. Yes   2. No   Other (please specify)  If yes, please provide details below for each service. |
| In practice, does your Trust accept patients aged 18 and above for treatment for their ADHD?   - 1. Yes   2. No   3. Other (please specify): |

| **Part 2: Service details - Service 1** |
| --- |
| Service 1 |
| 1. Name: 2. Town: 3. Website: 4. Service Main/Administrative Postcode: 5. Postcode/s of **all** locations where patients can access treatment: |
| Service type *(please indicate which and details if a specialist service)*:   1. Generic Adult Mental Health Service 2. Specialist Mental Health Service    1. ADHD    2. ADHD & ASD    3. ASD    4. Neurodevelopmental    5. Learning Disability    6. Other (*please provide details*): 3. Other (*please provide details*):   Ages served:   1. Upper age boundary? 2. Lower age boundary? |
| Adult ADHD Services *(please indicate)*:   1. Transitional Care   *(arrangements for transition of care from child to adult services)*   1. Diagnosis 2. Medication management   *(initial prescription, titration and/or monitoring & oversight)*   1. Ongoing prescribing of ADHD medication   *(provided directly by this service)*   1. Shared care   *(agreement with local physicians to prescribe, with monitoring by this service)*   1. Psychological treatment 2. Other, such as support groups…   *(please provide details)* |
| Commissioning:   1. Which Clinical Commissioning Groups (CCGs) commission this service?   *(names in full)*   1. Are patients from other CCGs or regions also able to access this service?   *Y/N? (If yes, please provide details)* |

| **Part 2: Service details - Service 2** |
| --- |
| Service 2 |
| 1. Name: 2. Town: 3. Website: 4. Service Main/Administrative Postcode: 5. Postcode/s of **all** locations where patients can access treatment: |
| Service type *(please indicate which and details if a specialist service)*:   1. Generic Adult Mental Health Service 2. Specialist Mental Health Service    1. ADHD    2. ADHD & ASD    3. ASD    4. Neurodevelopmental    5. Learning Disability    6. Other (*please provide details*): 3. Other (*please provide details*):   Ages served:   1. Upper age boundary? 2. Lower age boundary? |
| Adult ADHD Services *(please indicate)*:   1. Transitional Care   *(arrangements for transition of care from child to adult services)*   1. Diagnosis 2. Medication management   *(initial prescription, titration and/or monitoring & oversight)*   1. Ongoing prescribing of ADHD medication   *(provided directly by this service)*   1. Shared care   *(agreement with local physicians to prescribe, with monitoring by this service)*   1. Psychological treatment 2. Other, such as support groups…   *(please provide details)* |
| Commissioning:   1. Which Clinical Commissioning Groups (CCGs) commission this service?   *(names in full)*   1. Are patients from other CCGs or regions also able to access this service?   *Y/N? (If yes, please provide details)* |

| **Part 2: Service details - Service 3** |
| --- |
| Service 3 |
| 1. Name: 2. Town: 3. Website: 4. Service Main/Administrative Postcode: 5. Postcode/s of **all** locations where patients can access treatment: |
| Service type *(please indicate which and details if a specialist service)*:   1. Generic Adult Mental Health Service 2. Specialist Mental Health Service    1. ADHD    2. ADHD & ASD    3. ASD    4. Neurodevelopmental    5. Learning Disability    6. Other (*please provide details*): 3. Other (*please provide details*):   Ages served:   1. Upper age boundary? 2. Lower age boundary? |
| Adult ADHD Services *(please indicate)*:   1. Transitional Care   *(arrangements for transition of care from child to adult services)*   1. Diagnosis 2. Medication management   *(initial prescription, titration and/or monitoring & oversight)*   1. Ongoing prescribing of ADHD medication   *(provided directly by this service)*   1. Shared care   *(agreement with local physicians to prescribe, with monitoring by this service)*   1. Psychological treatment 2. Other, such as support groups…   *(please provide details)* |
| Commissioning:   1. Which Clinical Commissioning Groups (CCGs) commission this service?   *(names in full)*   1. Are patients from other CCGs or regions also able to access this service?   *Y/N? (If yes, please provide details)* |

| **Part 2: Service details - Service 4 onwards…** |
| --- |
| Please duplicate the forms above to provide details for as many mental health services as your trust/board provides for people with ADHD aged 18 years and above… |
