# Supplementary material for: Mapping UK mental health services for adults with attention-deficit/hyperactivity disorder: national survey with comparison of reporting between three stakeholder groups
Source: BJPsych Open. 2020 Jul 29;6(4):e76. doi: 10.1192/bjo.2020.65 (PMC7443899; doi:10.1192/bjo.2020.65)
Supplement: Supplementary file 1 [file S2056472420000654sup001.zip › S2056472420000654sup002.docx]

**Index**

1. Online survey
2. Key research partners
3. Freedom of information (FOI) request sent to UK organisations responsible for commissioning, to identify services for adults with ADHD (example)
4. FOI request sent to confirm details of treatment available at services identified in the mapping study (example)
