# Supplementary material for: Mapping UK mental health services for adults with attention-deficit/hyperactivity disorder: national survey with comparison of reporting between three stakeholder groups
Source: BJPsych Open. 2020 Jul 29;6(4):e76. doi: 10.1192/bjo.2020.65 (PMC7443899; doi:10.1192/bjo.2020.65)
Supplement: Supplementary file 1 [file S2056472420000654sup001.zip › S2056472420000654sup004.docx]

**Key Research Partners**

| **Name** | **Abbreviation** | **Website** |
| --- | --- | --- |
| AADD-UK The site for and by adults with ADHD | AADD-UK | <https://aadduk.org/> |
| ADHD Foundation |  | <https://www.adhdfoundation.org.uk/> |
| Association for Child and Adolescent Mental Health | ACAMH | <https://www.acamh.org/> |
| British Association for Community Child Health | BACCH | <http://www.bacch.org.uk/index.php> |
| Collaboration for Leadership in Applied Health Research and Care South West Peninsula | PenCLAHRC | <http://clahrc-peninsula.nihr.ac.uk/> |
| PenCRU: CATCh-uS Parent Advisory Group |  | <http://www.pencru.org/getinvolved/ourfamilyfaculty/> |
| Clinical Research Network, England | CRN | <https://www.nihr.ac.uk/about-us/how-we-are-managed/managing-centres/crn/> |
| Clinical Research Network South West Peninsula | CRN SW | <https://www.nihr.ac.uk/nihr-in-your-area/south-west-peninsula/> |
| Mental Health Commissioners Network | MHCN | <https://www.nhscc.org/networks/mental-health-commissioners/> |
| Royal College of General Practitioners | RCGPs | <http://www.rcgp.org.uk/> |
| Royal College of Psychiatrists | RCPsych | <https://www.rcpsych.ac.uk/> |
| UK Adult ADHD Network | UK-AAN | <https://www.ukaan.org/> |
