# Supplementary material for: Mapping UK mental health services for adults with attention-deficit/hyperactivity disorder: national survey with comparison of reporting between three stakeholder groups
Source: BJPsych Open. 2020 Jul 29;6(4):e76. doi: 10.1192/bjo.2020.65 (PMC7443899; doi:10.1192/bjo.2020.65)

**Subject**: Freedom of information request - CATCh-uS 2018 National survey mapping adult ADHD services

**Text:**

Dear Sir/Madam

Please provide the following information about Attention Deficit Hyperactivity Disorder (ADHD) services in your area for people aged 18 and above. Alternatively forward to the relevant mental health commissioning lead to answer.

*NB if your Clinical Commissioning Group (CCG) does not provide mental health services, please provide details of the CCG that covers these services for your area.

**If you provide FOI for more than one CCG, please answer questions separately for **every** CCG you represent – or once for the new ‘merged’ CCG you represent. This is a national survey so we want to know about all CCGs and we have only contacted FOI email addresses once, to avoid duplication of workload.

Thank you in advance for your support.

**2018 Survey for the ‘Children and adolescents with Attention Deficit Hyperactivity Disorder in transition between children’s services and adult services’ (CATCh-uS) study**

The CATCh-uS study is funded by the National Institute for Health Research and has ethical approval. Details can be found on our [website](http://medicine.exeter.ac.uk/catchus/) (<http://medicine.exeter.ac.uk/catchus/>). The aim of this survey is to map available services for people with ADHD who are aged 18 and over. This is to inform and improve service provision for young adults with ADHD. Answers will help to update a list of existing services, available [here](http://medicine.exeter.ac.uk/catchus/mapping/adhdservices/) (<http://medicine.exeter.ac.uk/catchus/mapping/adhdservices/>).

1. Which NHS Clinical Commissioning Group/s (CCG) do you represent?
   1. Name:
   2. Postcode:
2. Please provide details of the Mental Health Commissioner (lead) for your CCG (the person responsible for commissioning adult ADHD services).
   1. Name:
   2. Email Address:
   3. Phone Number:
   4. Job Role:
3. Your details:
   1. Name:
   2. Email Address:
   3. Phone Number:
   4. Job Role:
4. Please indicate which NHS England region your CCG is part of:
   1. London
   2. Midlands and East of England
   3. North of England
   4. South of England
5. Does your CCG commission mental health services that treat/support people with ADHD aged 18 years and above?
   1. Yes
   2. No
6. Please provide details of the service/s (for as many services as you have)
   1. Name:
   2. Postcode:
   3. Town:
   4. Website:
   5. This service is a part of (please indicate):
      1. Adult Mental Health Services
      2. Child and Adolescent Mental Health Services
      3. A Specialist Mental Health Service
      4. Other
      5. Don't know
   6. This service offers (please indicate):
      1. Treatment (Medication)
      2. Treatment (Other Intervention)
      3. Assessment
      4. Diagnosis
      5. Other
      6. Don’t know

Thank you for providing this information. If you would like further information about this NIHR funded study please access our [website](http://medicine.exeter.ac.uk/catchus/mapping/). Thank you for your participation!

Best wishes,


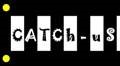
Anna

**Anna Price**

Associate Research Fellow

01392 726026

<http://medicine.exeter.ac.uk/catchus/>

CATCh-uS – Children and Adolescents with ADHD in transition from Children Services to Adults Services.

University of Exeter Medical School, South Cloisters (room 2.05), St Luke's Campus, Exeter EX1 2LU.


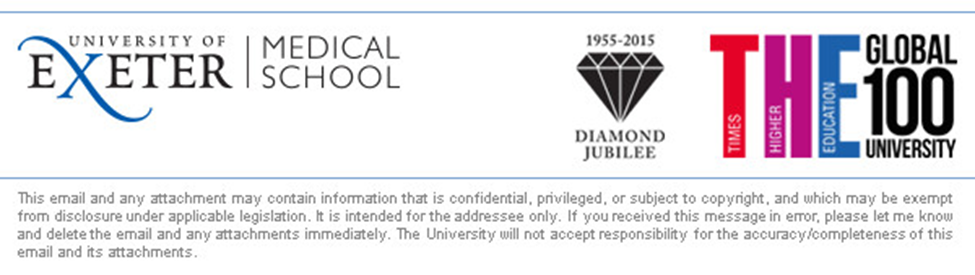

Supplement: Supplementary file 1 [file S2056472420000654sup001.zip › S2056472420000654sup005.docx]
